# LL-37 Triggers Antimicrobial Activity in Human Platelets

**DOI:** 10.3390/ijms24032816

**Published:** 2023-02-01

**Authors:** Francisco Javier Sánchez-Peña, María de los Ángeles Romero-Tlalolini, Honorio Torres-Aguilar, Diego Sait Cruz-Hernández, Rafael Baltiérrez-Hoyos, Saraí Remedios Sánchez-Aparicio, Alba Soledad Aquino-Domínguez, Sergio Roberto Aguilar-Ruiz

**Affiliations:** 1 Departamento de Biomedicina Experimental, Facultad de Medicina y Cirugía de la Universidad Autónoma “Benito Juárez” de Oaxaca, Oaxaca 68120, México; 2 Consejo Nacional de Ciencia y Tecnología, Facultad de Medicina y Cirugía de la Universidad Autónoma “Benito Juárez” de Oaxaca, Oaxaca 68120, México; 3 Facultad de Ciencias Químicas, Universidad Autónoma “Benito Juárez” de Oaxaca, Oaxaca 68120, México

**Keywords:** platelets, LL-37, antimicrobial molecules, receptors for microorganism recognition

## Abstract

Platelets play a crucial role in hemostasis and the immune response, mainly by recognizing signals associated with vascular damage. However, it has recently been discovered that the antimicrobial peptide LL-37 activates platelets in functions related to thrombus formation and inflammation. Therefore, this work aims to evaluate the effect of LL-37 on the activation of antimicrobial functions of human platelets. Our results show that platelets treated with LL-37 increase the surface expression of receptors (Toll-like receptors (TLRs) 2 and -4, CD32, CD206, Dectin-1, CD35, LOX-1, CD41, CD62P, and αIIbβ3 integrins) for the recognition of microorganisms, and molecules related to antigen presentation to T lymphocytes (CD80, CD86, and HLA-ABC) secrete the antimicrobial molecules: bactericidal/permeability-increasing protein (BPI), azurocidin, human neutrophil peptide (HNP) -1, and myeloperoxidase. They also translate azurocidin, and have enhanced binding to Escherichia coli, Staphylococcus aureus, and Candida albicans. Furthermore, the supernatant of LL-37-treated platelets can inhibit E. coli growth, or platelets can employ their LL-37 to inhibit microbial growth. In conclusion, these findings demonstrate that LL-37 participates in the antimicrobial function of human platelets.

## 1. Introduction

Platelets are small, anucleate, discoid cells, ranging in size from 2 to 5 µm. After erythrocytes, they are the most abundant in the blood, with values of 150–450 × 10^9^/L. Once released from their megakaryocytic precursors, platelets enter the bloodstream, where they can circulate for seven to ten days [1]. These cells present several organelles, including α-granules, dense granules, peroxisomes, lysosomes, mitochondria, and a dense tubular system involved in Ca^2+^ sequestration, which participates in intracellular signaling cascades [2]. Moreover, transcriptional analyses indicate that platelets contain thousands of messenger RNAs (mRNAs) [3] and a functional spliceosome that allows for the processing of interleukin (IL)-1β, platelet factor XI (FXI), tissue factor (TF), and cyclooxygenase (COX)-2 transcripts [4]. In addition, platelets perform basal [5,6] and activation-inducible translation [7,8].

The primary described function of platelets is hemostasis. In this process, express glycoproteins (GPVI and GPIbα) and integrins (α_2_β_1_, α_IIb_β_3_, α_V_β_3_, and α_5_β_1_) enable them to detect vascular damage by recognizing subendothelial components, including collagen fibers, the von Willebrand factor (vWF), and fibronectin, present in this condition. This further leads to platelet activation and the development of a hemostatic clot, formed by platelet aggregation and fibrin deposits (the result of the coagulation cascade), to prevent blood loss [9,10]. Furthermore, to preserve vascular integrity, human platelets have an essential role in the immunological response, with their repertoire of antimicrobial molecules including azurocidin [11], kinocidins, CXC chemokine ligand (CXCL)-4, CXCL7, CCL5, and most of the cationic host defense peptides (CHDPs), which correspond to the human neutrophil peptide (HNP)-1, human beta-defensins (HBD)1-3, and hCAP18 cathelicidin (18 kDa human cationic antimicrobial peptide) [12].

hCAP18 is expressed by different cell types and comprises epithelial cells, keratinocytes, neutrophils, natural killer (NK) cells, dendritic cells, monocytes/macrophages, lymphocytes, mast cells, mesenchymal stem cells, and bone marrow stroma. Once hCAP18 is released from its intracellular environment, it is processed by specific proteases into the active form of the LL-37 peptide, which has a wide range of antibacterial, antifungal, and antiviral activity [13,14]. In addition, LL-37 is chemoattractant, immunomodulatory, and pro- or antitumor, which depends on the direct or indirect recognition of LL-37 by one of its different receptors on its target cells, which include FPR2 (N-formyl peptide receptor 2), CXC motif chemokine receptor (CXCR)-2, MrgX2 (Mas-related gene X2), P2RY11 (purinergic receptor P2Y11), EGFR (epidermal growth factor receptor), and TLRs (Toll-like receptors)-3 and -4 [15]

The treatment of platelets with LL-37 or with the peptide itself, released by platelets after stimulation, plays a central role in their activation, causing different effects that involve intracellular Ca^2+^ mobilization, aggregation, thrombus formation, CD62P expression, and adenosine triphosphate (ATP) release; these effects are inhibited by selectively blocking FPR2 [16]. In a similar context, LL-37 is found in coronary artery thrombi from patients, and because the hemostatic activation of platelets is linked to their immune activity, treating human platelets with LL-37 leads to an increased secretion of CD40L and IL-1β and favors the formation of platelet–neutrophil aggregates [17]. Furthermore, mouse platelets treated with CRAMP (a molecule orthologous to LL-37) and transferred to a murine model of trauma-induced inflammation promote neutrophil recruitment and extravasation [17]. HNP1 and LL-37 are the only CHDPs that have been shown to activate human platelets, although knowledge of the involvement of LL-37 in antimicrobial function in platelets is null. Similarly, treating platelets with HNP1 leads to platelet activation, elevating parameters related to hemostatic function and inflammation, and authors have shown that platelets form myeloid-like structures which allow them to capture *S. aureus* and *C. albicans* [18]. Therefore, our objective in this investigation was to evaluate the involvement of the LL-37 parameters of platelet antimicrobial activity corresponding to immunophenotyping, release, and translation of antimicrobial molecules, as well as the binding and elimination of microorganisms.

## 2. Results

### 2.1. LL-37 Increases the Surface Expression of Receptors for Recognizing Microorganisms

**Figure 1:**
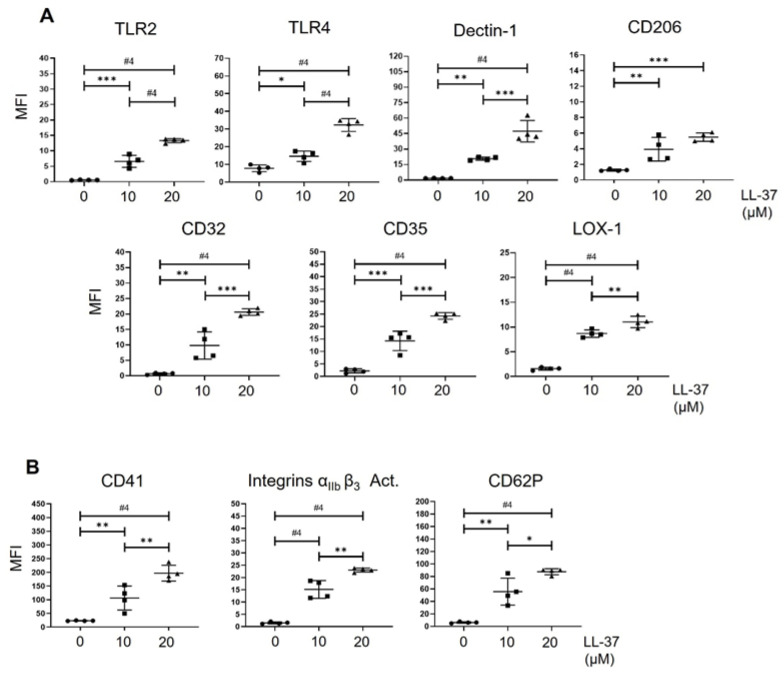
**Platelets increase the expression of receptors for recognizing microorganisms in response to LL-37**. Platelets were purified as described in the materials and methods, treated with LL-37 (10 or 20 µM of LL-37) or left peptide-free (control) over 30 min at 37 °C and 5% CO_2_. Subsequently, platelets were stained using monoclonal antibodies (mAbs) against each of the receptors of the innate immune response (**A**) or hemostasis (**B**) related to the recognition of microorganisms. Each graph represents the mean ± standard deviation of four independent experiments’ mean fluorescence intensity (MFI). Statistical significance was determined by one-way ANOVA and is expressed as * *p* < 0.05, ** *p* < 0.01, *** *p* < 0.001, and #4 *p* < 0.0001.

Initially, we determined the impact of LL-37 on the expression of receptors related to the direct or indirect recognition of microorganisms on human platelets. The receptors evaluated were TLRs-2 and -4, the low-affinity receptor for the crystallizable fraction of immunoglobulin (Ig) G (FcγR11a/CD32), C-type lectin domain family 7, member A (dectin 1), complement receptor type 1 (CR1/CD35), mannose receptor (CD206), and lectin-like oxidized low-density lipoprotein receptor 1 (LOX-1). The concentrations of 10 and 20 µM LL-37 reported by other investigators [16,17] were used for a 30 min stimulation time. Our results indicated that all the receptors evaluated increased their expression on the platelet surface after treatment with both concentrations of LL-37 (Figure 1A).

On the other hand, it is known that platelets also use their hemostatic receptors to recognize microorganisms directly or indirectly. These receptors are CD41(integrin α_IIb_), the α_IIb_β_3_ integrins in their active conformation, and CD62P [19,20]. Therefore, we evaluated their expression and found that platelets present higher levels of these receptors when treated with both concentrations of LL-37 (Figure 1B). In conclusion, we found that platelets treated with LL-37 increase their expression of receptors that could allow for a higher recognition of microorganisms.

### 2.2. LL-37-Treated Platelets Demonstrate Increased Binding to Microorganisms

**Figure 2:**
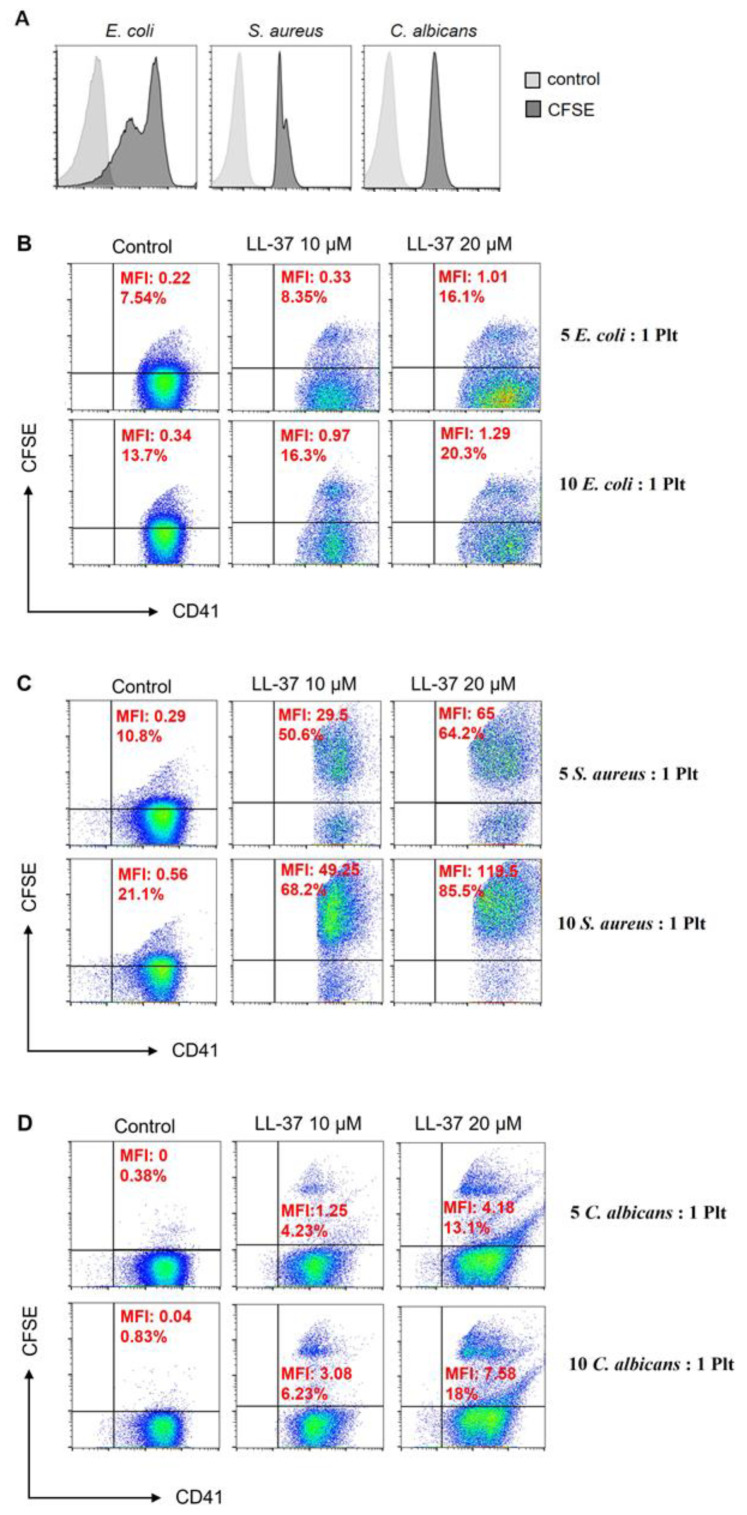
**Platelets treated with LL-37 have a more significant interaction with microorganisms**. (**A**) *E. coli*, *S aureus*, and *C. albicans* microorganisms were stained with CFSE. In histograms, the dark gray represents staining with CFSE, and the light gray color represents microorganisms without CFSE. CFSE-stained microorganisms were placed in contact with platelets treated with 10 or 20 µM of LL-37 for 3 h and then analyzed by flow cytometry. The dot plot comes from the gate of CD41^+^ platelets (x-axis) and their interaction with *E. coli*-CFSE (**B**), *S. aureus*-CFSE (**C**), and *C. albicans*-CFSE (**D**) (y-axis). To the right of Figures (**B**–**D**) are indicated the relationships between microorganisms and platelets. The upper right quadrant shows the percentage and MFI of platelet interaction with each microorganism. The figure is representative of four independent experiments.

Once we found that treatment of platelets with LL-37 increased the expression of receptors related to the recognition of microorganisms, we stained *E. coli* and *S. aureus* bacteria and the yeast *C. albicans* with a fluorescent tracer, 5(6)-carboxyfluorescein diacetate N-succinimidyl ester (CFSE) (Figure 2A), and contacted them with platelets treated with 10 and 20 µM of LL-37 or with untreated platelets as a control. The results indicated that platelets stimulated with LL-37 increased the formation of platelet–*E. coli* and platelet–*C. albicans* interactions (Figure 2B,D). However, platelet–*S. aureus* interactions were the highest with our experimental conditions (Figure 2C). We found that a higher ratio of microorganisms (or the concentration of LL-37) led to a more significant interaction between platelets and microorganisms.

### 2.3. LL-37 Triggers the Release of Antimicrobial Molecules in Human Platelets

**Figure 3:**
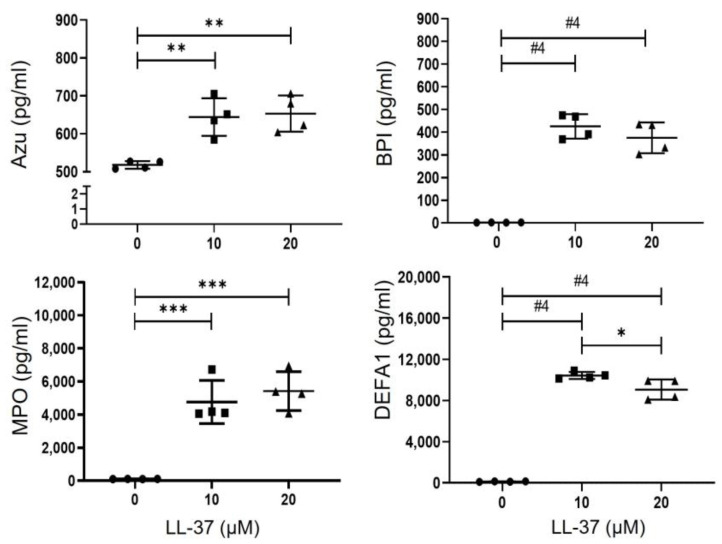
**Platelets secrete antimicrobial molecules in response to LL-37**. 450 × 10^6^ platelets/mL were treated with 10 or 20 µM of LL-37 for 30 min at 37 °C and 5% CO_2_ or without LL-37 as a control. Subsequently, the supernatant was obtained and used to quantify the concentration of Azu, BPI, MPO, and HNP1 by ELISA. The graphs represent the mean ± standard deviation of four independent experiments. Statistical significance was determined by one-way ANOVA and denoted as * *p* < 0.05, ** *p* < 0.01, *** *p* < 0.001, and #4 *p* < 0.0001.

Subsequently, we evaluated the effect of LL-37 on the release of molecules with antimicrobial activity. We stimulated platelets with 10 or 20 µM of LL-37 for 30 min, and the supernatant was used to evaluate the presence of azurocidin (Azu), BPI (bactericidal/permeability-increasing protein), HNP-1, and MPO (myeloperoxidase). Our results indicated that LL-37 treatment increased the secretion of all antimicrobial molecules evaluated. Azu (518 ± 10.18 pg/mL vs. 644.3 ± 49.65 pg/mL and 653.4 ± 47.8 pg/mL), BPI (1.52 ± 0.41 pg/mL vs. 425.5 ± 54.12 pg/mL and 375.2 ± 67.8 pg/mL), MPO (144. 6 ± 4.0 vs. 2422 ± 612.3 pg/mL and 2732 ± 468.7 pg/mL), and HNP-1 (130.9 ± 25.65 pg/mL vs. 10434 ± 336.8 pg/mL and 9068 ± 977.7 pg/mL) (Figure 3).

### 2.4. LL-37 Impacts the Antimicrobial Response of Platelets

#### 2.4.1. Platelet LL-37 Inhibits Microbial Growth

**Figure 4:**
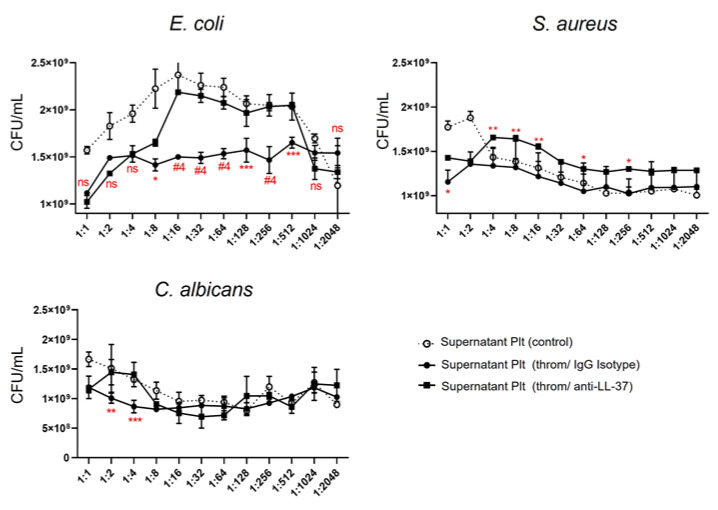
**Platelet LL-37 inhibits microbial growth**. Platelets were purified according to materials and methods to be stimulated with thrombin (throm) or no stimulation (control) for 30 min at 37 °C. The supernatant of activated platelets was treated with an isotype IgG mAb or an anti-LL-37, neutralizing mAb for 30 min at 5 °C before contact with each of the microorganisms indicated in the figure. The number of colony-forming units (CFU, y-axis) was evaluated by broth microdilution assay for 13 h and calculated from the optical density by the McFarland method. Statistical significance was determined by one-way ANOVA and denoted as * *p* < 0.05, ** *p* < 0.01, *** *p* < 0.001, and #4 *p* < 0.0001, and corresponds to the comparison of throm/IgG isotype vs. the throm/anti-LL-37 condition. The graphs represent the mean ± standard deviation of three independent experiments of the effect of the platelet supernatant and their dilutions used (x-axis) with each of the treatments described.

In addition to responding to exogenous LL-37, platelets contain and secrete LL-37 upon stimulation [16]. We asked ourselves what the contribution of platelet LL-37 in the antimicrobial response was. To this end, platelets were stimulated with thrombin for 30 min, and the platelet supernatant was treated with a neutralizing mAb or an isotype control mAb and placed in contact with growing *E. coli*, *S. aureus*, and *C. Albicans* for 13 h. Our results showed that platelet LL-37 inhibits microbial growth, mainly against *E. coli*, because the neutralizing LL-37 mAb allows for a significant recovery of them (Figure 4).

#### 2.4.2. The Platelet Supernatant Treated with LL-37 Inhibits the Growth of *E. coli*

**Figure 5:**
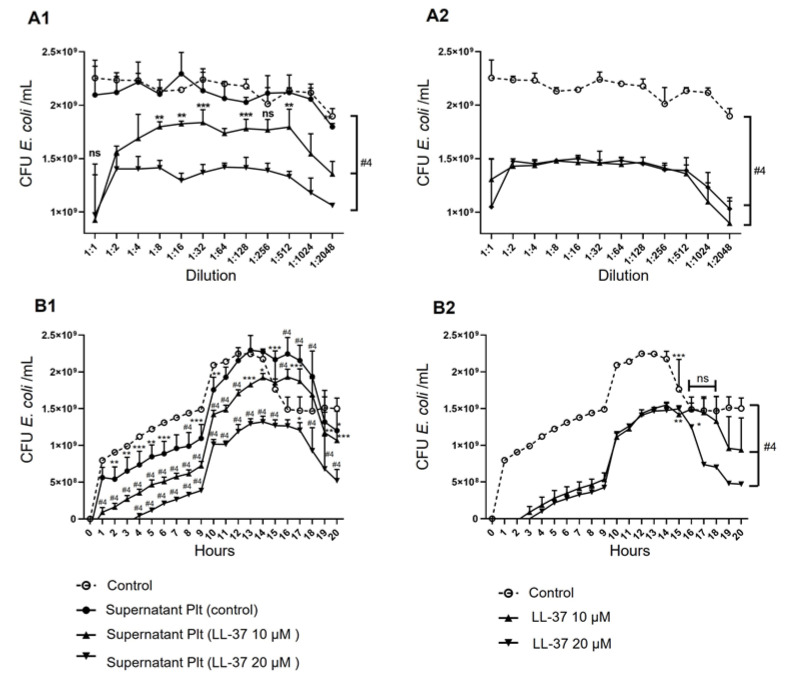
**The supernatant of LL-37-treated platelets exhibits antibacterial activity against *E. coli.*** 450 × 10^6^ platelets/mL were treated with 10 or 20 µM LL-37 or untreated for 30 min. The supernatants obtained from activated platelets and LL-37 alone were used for their inhibitory effect on *E. coli* growth from the broth microdilution assay for 20 h. The graphs represent the average ± standard deviation of four independent experiments on the effect of dilutions of platelet supernatants with and without LL-37 treatment (**A1**) and LL-37 alone (**A2**), at 13 h, or as 1:16 dilutions over time ((**B1**) and (**B2**)), on the number of *E. coli* CFU (y-axis) calculated from the optical density by the McFarland method. Statistical significance was determined by one-way ANOVA and denoted as * *p* < 0.05, ** *p* < 0.01, *** *p* < 0.001, and #4 *p* < 0.0001.

Once we found that LL-37 induces the release of antimicrobial molecules, we proposed that the supernatant of LL-37-treated platelets could inhibit the growth of *E. coli*. We decided to include the effect of LL-37 itself due to its known bactericidal effect. We employed the untreated (control) or treated supernatants with 10 and 20 µM of LL-37 with *E. coli* bacteria. The results showed that, after 13 h of growth, different dilutions (1:1 to 1:2048) of the supernatant of LL-37-treated platelets decreased the number of colony-forming units (CFU) of *E. coli*, mainly when stimulated with 20 µM. In contrast, the supernatant of the untreated platelets seemed to have a minimal effect (Figure 5A1). Similar results were found when analyzing *E. coli* CFU over time (1–24 h), using 1:16 dilutions of the platelet supernatant (Figure 5B1). However, LL-37 (10 or 20 µM) alone, evaluated at the same dilutions and times as the platelet supernatants, inhibited *E. coli* growth to a magnitude akin to the secretion product of the LL-37-treated platelets (Figure 5A2,B2). Therefore, these results indicate that the supernatant of LL-37-treated platelets inhibits the growth of *E. coli*. However, its effect may result from the own antimicrobial molecules released by the platelets (Section 2.3), with the LL-37 added as a platelet stimulus in the supernatant.

### 2.5. LL-37 Induces Translation of Azurocidin in Platelets

**Figure 6:**
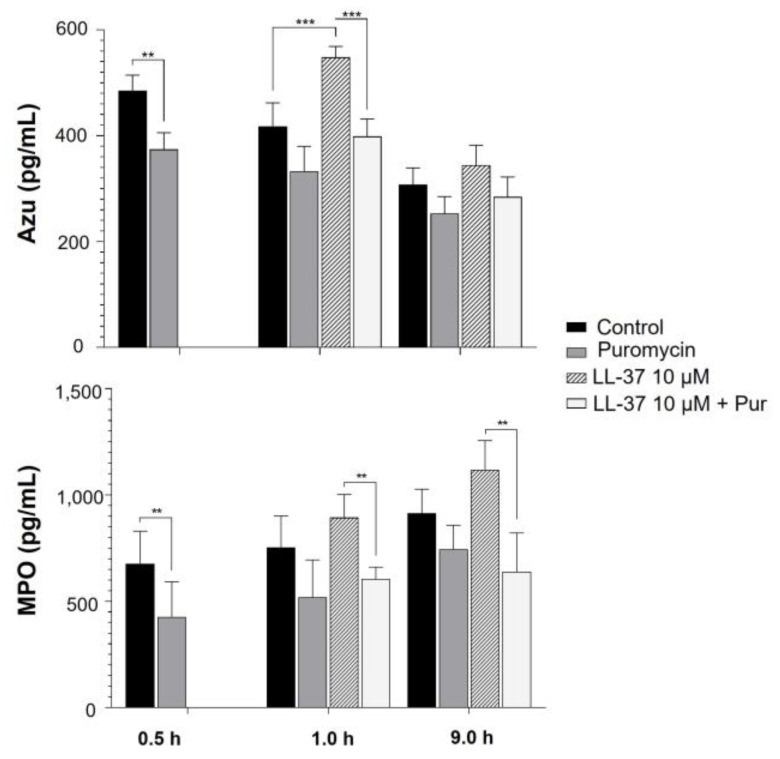
**LL-37 induces azurocidin translation in platelets**. 120 × 10^6^ platelets were treated with 10 µM of puromycin (Pur) and were stimulated with LL-37 0.5 h later. Untreated platelets were used as controls. Subsequently, total protein (cell and supernatant) was obtained at 0.5 h (1 h total: 0.5 h with puromycin + 0.5 h with LL-37 stimulation), and 9 h after stimulation. Subsequently, the concentrations of Azu and MPO were quantified by ELISA. In the figure, the graphs represent the mean ± standard deviation of four independent experiments. Statistical significance was determined by a 2-way ANOVA test with repeated measures and is represented as ** *p* < 0.01, and *** *p* < 0.001.

Further, to determine whether LL-37 can induce the translation of other antimicrobial molecules, we decided to test its effect on Azu translation, since the antimicrobial protein azurocidin has recently been demonstrated in thrombin-treated human platelets [11]. Additionally, we used this opportunity to evaluate the translation of MPO induced by LL-37 because, until now, there was no knowledge about its translation in human platelets. Therefore, platelets were treated with a translation inhibitor (puromycin) for 0.5 h and subsequently activated with LL-37 for 0.5 h and 9 h. The total azurocidin and MPO (cell/supernatant) concentrations were measured by ELISA. The results indicated that the concentration of azurocidin in the platelets decreased in culture over time, with basal translation found to occur mainly at 0.5 h. Platelets treated with LL-37 for 0.5 h led to activation-induced azurocidin translation, as puromycin treatment inhibits this effect (Figure 6). Similarly, the contribution of basal MPO translation occurred at 0.5 h. However, unlike Azu, the basal (control) MPO concentration did not decay over time, the activation of platelets with LL-37 increased on average the MPO concentration (but not significantly), and the puromycin treatment of the LL-37-treated platelets decreased the MPO concentration below the control condition, indicating that both basal and induced translation was inhibited (Figure 6).

### 2.6. LL-37 Increases Platelet Surface Expression of Molecules for Antigen Presentation to T Lymphocytes

**Figure 7:**
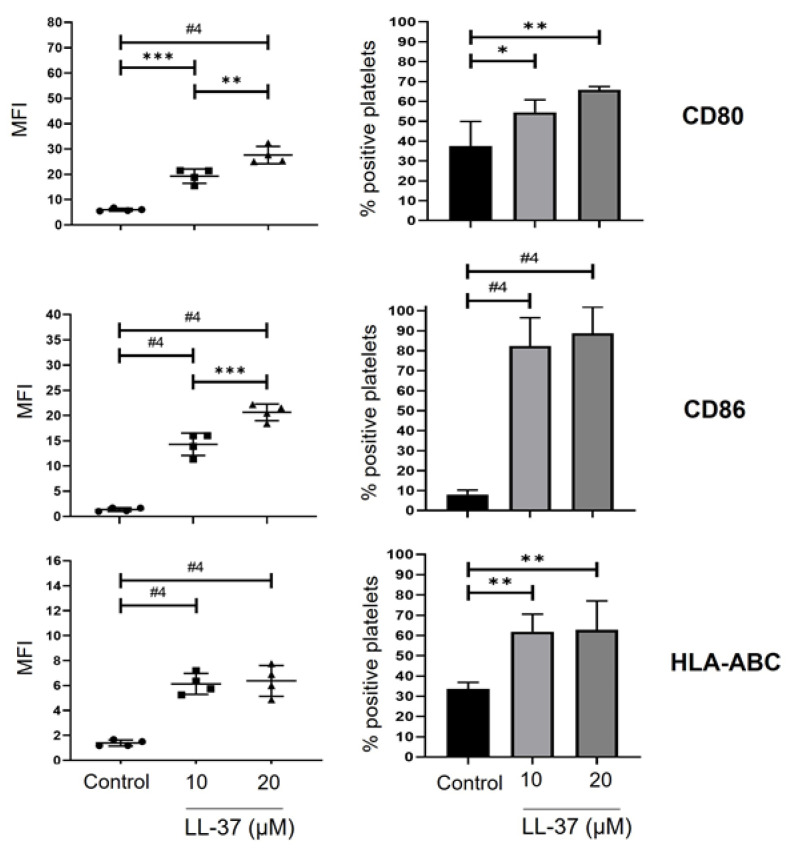
**LL-37-treated platelets increase the expression of antigen-presenting molecules**. Platelets purified as described in materials and methods were treated with 10 or 20 µM LL-37 or no treatment (control) for 30 min at 37 °C and 5% CO_2_. Subsequently, platelets were stained using mAb (monoclonal antibody) against CD41, CD80, CD86, and HLA ABC. Each graph represents the mean ± standard deviation of the mean fluorescence intensity (MFI) the total CD41+ platelet population or the percentage of positive platelets from four independent experiments. All results are from the preselection of the CD41^+^ platelet population. Statistical significance was determined by one-way ANOVA and is represented as * *p* < 0.05, ** *p* < 0.01, *** *p* < 0.001, and #4 *p* < 0.0001.

Finally, we considered that another way in which the LL-37-activated platelets could influence antimicrobial defence is through the activation of the adaptive immune response. Therefore, we evaluated the expression of molecules related to antigen presentation to T lymphocytes. The results demonstrated that platelets treated with 10 or 20 µM LL-37 significantly increased their surface expression. Additionally, the percentage of platelets positive for the co-stimulators CD80 and CD86, in addition to the human leukocyte antigen (HLA) ABC, also known as major histocompatibility complex (MHC) class I (Figure 7). An example of our analysis is shown in Supplementary Figure S1.

## 3. Discussion

Nowadays, LL-37 is known to activate the hemostatic functions of platelets [16], together with immune functions, an increased expression of CD62P, the secretion of sCD40L and IL-1β, and the activation of neutrophils from the formation of aggregates with these cells [17]. However, the impact of LL-37 on the microbicidal function of platelets remained unknown; therefore, we conducted this research.

The 10 and 20 µM concentrations of LL-37 used to treat platelets in this study have been used by other authors [16,17] and are present in tissues from psoriasis patients (up to 300 µM) [21] and in tracheal aspirates from infected infants (1–10 µM) [22]. Our results indicate that LL-37 increases the platelet surface expression of receptors related to microorganism recognition directly (TLR2, TLR4, Dectin 1, CD209, and LOX-1) or indirectly (CD35 and CD32). In addition, LL-37 increases the expression of receptors with a hemostatic function, which platelets use to recognize microorganisms (CD41, the activated α_IIb_β_3_ integrins, and CD62P). The presence of these receptors is widely known in platelets [19,20]. However, regarding Dectin-1 and CD206, this is the first report of their presence on the platelet surface. The up-regulation of TLR4, TLR2, CD62P, and CD32 has been described in sepsis and is associated with an increased ability to bind *E. coli* bacteria [23]. The rapid increase of receptors on the platelet surface may be due to their mobilization from granule-α to their activation-induced translocation on the cytoplasmic membrane; a mobilization demonstrated for TLR4 [24], CD62P, and CD41 [16,17,25].

The increased immunoreceptors on the platelet surface prompted us to consider a greater capacity for binding microorganisms. We verified this by finding that LL-37-treated platelets increase their interaction with *S. aureus* and, to a lesser extent, with *C. albicans* and *E. coli.* Platelet interaction with *S. aureus* indirectly occurs when the protein A of this bacterium interacts with fibrinogen or the von Willebrand factor and integrins α_IIb_β_3_ and GP1bα subsequently recognize them, respectively, present in the platelets [26]. On the other hand, TLR2 is of great relevance for detecting *S. aureus* [27]. However, the involvement of this receptor in platelets is still unknown. In the case of platelet interaction with *E. coli*, one theory of how it occurs is through CD32, integrin α_IIb_β_3,_ and TLR4 receptors, using in, most cases, platelet-rich plasma [28]. Other receptors, such as LOX-1, recognize Gram-negative and Gram-positive bacteria; however, their role in detecting microorganisms on platelets is unknown. [29]. In contrast, it is better known that the α_IIb_β_3_ integrin plays a central role in recognizing *C. albicans* [30]. In addition, Dectin-1 and CD206, identified on platelets in this study, could have a relevant role in binding *C. albicans* based on the background of these receptors [31,32]. Therefore, it is essential to identify the contribution of each receptor expressed on platelets after the activation with LL-37 in binding to microorganisms.

In addition to an enhanced interaction with microorganisms, our findings show that LL-37-treated platelets secrete Azu, BPI, MPO, and HNP1. Furthermore, we have also recently found that thrombin, ADP (Adenosine Diphosphate), and Lipopolysaccharide (LPS) induce the secretion of Azu and HNP1 in platelets [11,33]. Thus, this is the first description of BPI and MPO secretion by human platelets. In addition to the molecules evaluated, it is suggested that others, including CXCL4, CXCL7, CCL5, and HBD1-3, could be secreted [12]. These results led us to consider that the secretion product of platelets treated with LL-37 had a more significant antimicrobial effect than LL-37 alone. However, results obtained indicate that the antimicrobial function is similar between platelet supernatants and LL-37 alone, so we suggest that the observed effect is a combination of antimicrobial molecules secreted by LL-37-activated platelets (e.g., Azu, BPI, MPO, and HNP1) in addition to the LL-37 itself, used as a stimulus. We believe that the most appropriate way to discern this effect is by using a truncated or mutated LL-37, with the ability to activate its cellular receptor without triggering its antimicrobial activity. In this sense, the amino acids of LL-37 needed to activate its FPR2 receptor are already known [16]. In addition, we found that thrombin-activated platelets employ their LL-37 to inhibit the growth of *E. coli, S aureus*, and *C. Albicans*. This had not been previously described and becomes part of the repertoire of antimicrobial molecules that platelets secrete as part of their defense mechanisms [12,34].

Our results also show that LL-37 triggers the translation of the antimicrobial protein Azu at early times (30 min), while MPO translation occurs mainly in a basal manner. Previously, our research group demonstrated the translation of Azu with thrombin [11]. We suggest that it is relevant to know the impact of LL-37 on the translation of other molecules with antimicrobial and inflammatory activity and for whom the mRNA is also present [35]. Furthermore, precedents demonstrate that both mouse and human platelets express co-stimulatory molecules and HLA-ABC (MHC class I) [36,37,38,39], and, at least in mouse platelets, they can activate T lymphocytes both in vitro and in vivo [38]. We found that human platelets treated with LL-37 increased the expression of co-stimulators CD80 and CD86 and HLA-ABC, suggesting that LL-37 could induce an antimicrobial function of platelets by increasing their antigen-presenting cell (APC) capacity or allow platelets to contribute co-stimulators to APCs for T lymphocyte activation. However, the involvement of human platelets in antigen presentation remains to be evaluated.

The findings described in this article are relevant because, until now, thrombin, ADP, and LPS were mainly recognized as the stimuli that induce the secretion of microbicidal molecules [12,34]. However, in this work, LL-37 is also shown to trigger this activity in platelets and other functions. Finally, it is known how LL-37 carries out its effects on platelets upon recognition by the FPR2 receptor, and how it triggers the production of cyclic adenosine monophosphate (cAMP) [16]. However, other authors demonstrate that the receptor responsible for the effects of LL-37 is glycoprotein (GP) VI, which leads to the activation of Syk tyrosine kinases, Src family kinases, STAT3 (signal transducer and activator of transcription 3), and intracellular Ca^2+^ release [17].

## 4. Materials and Methods

### 4.1. Purification of Platelets from Peripheral Blood

For this study, we used blood obtained from clinically healthy donors, with aspirin or drugs that could affect platelet function discontinued at least two weeks before sample collection. Blood was co-collected from the antecubital vein using Vacutainer tubes with ACD (acid-citrate-dextrose) (BD Biosciences, Franklin Lakes, NJ, USA). We obtained platelet-rich plasma (PRP) by centrifuging the blood at 250× *g* for 15 min (Thermo Scientific Sorvall ST 8R centrifuge, New District, Suzhou, Jiangsu, China). We sought purification efficiency by recovering two-thirds of the top layer of PRP, transferring it to a new, sterile plastic tube, and centrifuging it at 150× *g* for 20 min at room temperature (using slow deceleration). The resulting supernatant was transferred to a new tube and centrifuged at 2200× *g* for 15 min at room temperature to precipitate the platelets. The precipitate was washed twice by adding 3 mL of Tyrode buffer with ACD (1:6 *v*/*v*). The washes were centrifuged at 1900× *g* for 8 min to precipitate platelets. For either assay, the platelet pellet was resuspended at a final density of 450 × 10^6^ cells/mL in Tyrode buffer supplemented with 0.01 U/mL of apyrase (Sigma-Aldrich, St. Louis, MO, USA) to prevent activation.

Platelet purity was verified by light microscopy (Microscope Swift, Model M17B-MP, Hicksville, NY, USA) and flow cytometry, the second using mAb APC (allophycocyanin) mouse anti-human CD11b (clone: D12), and PE-Cy5 mouse anti-human CD41 (clone: HIP8) and the isotype controls APC mouse IgG2a, κ isotype (clone: G155-178), and PE-Cy5 Mouse IgG1 κ Isotype (clone: MOPC-21), all from BD Biosciences (Franklin Lakes, NJ, USA). Finally, platelets were fixed with 4% paraformaldehyde for 30 min and acquired in a MACSQuant flow cytometer (Milteny Biotec, Bergisch Gladbach, Germany). The obtained data were analyzed using FlowJo software Version 10 (FlowJo, Ashland, OR, USA).

### 4.2. Effect of LL-37 on the Immunophenotype of Peripheral Blood Platelets

A total of 400 × 10^6^ platelets, purified as described in Section 4.1, were suspended in 1 mL of Tyrode’s buffer and stimulated with 10 or 20 µM of LL-37 (Tocris Bioscience, Bristol, UK) for 20 min at 37 °C in a 5% CO_2_ atmosphere (Incubator NUAIRE, model NU-5700, Plymouth, MN, USA). After platelet simulation, the cells were obtained by centrifugation at 480 g for 10 min and the supernatant was stored at −70 °C. Subsequently, platelets were stained using mouse mAb directed against the following human molecules: anti-CD41-PE-Cy5 (Section 4.1), anti-HLA-ABC-APC (clone: G46-2.6), anti-CD86-FITC (2331 (FUN-1)), anti-CD282-APC (clone: 11G7), anti-CD32-APC (clone: FLI8. 26), anti-CD35-FITC (clone: E11) (BD Biosciences, USA), anti-PAC-1-FITC (clone: PAC-1), an-ti-CD62P-FITC (clone: AK4), anti-LOX-1-PE (clone: 15C4), anti-CD80-FITC (clone: 2D10) (Biolegend, San Diego, CA, USA), anti-CD284-APC (clone: HTA125) (Invitrogen, Waltham, MA, USA), anti-PAR1-PE (clone: 731115) (R & D System, Minneapolis, MN, USA), and anti-Dectin1-PE (clone: REA515) (Miltenyi Biotec, San Francisco, CA, USA), and using their respective mouse isotype mAbs: PE-Cy5 IgG1 κ Isotype (Section 4.1), APC IgG1 κ (clone: MOPC-21), APC IgG2b κ (clone: 27-35), FITC IgG1 κ Isotype (clone: MOPC-21), PE IgG1 κ (clone: X40), FITC IgM, κ (clone: MM-30) (Biolegend, San Diego, CA, USA), and PE IgG2b (clone: 133303) (R and D System, USA).

### 4.3. Platelet-Microorganism Interaction Assays

To determine the ability of platelets to interact with microorganisms, we selected the bacterial strains *E. coli* (ATCC 25922) and *S. aureus* (ATCC 29213) and the yeast *C. albicans* (ATCC 10231). The interaction was measured using the fluorescent marker CFSE (Thermo Fisher Scientific, Waltham, MA, USA) and the anti-human CD41 antibody coupled to PE-Cy5 (Section 4.1). Microorganisms were stained with 20 µM of CFSE for 3 h at 4 °C, and platelets were stained with anti-CD41 for 30 min at 4 °C. They were then fixed in 1% paraformaldehyde and set to interact in a Tyrode buffer with constant shaking at 120 rpm for 3 h at 4 °C in the ratios of 1:50, 1:25, 1:10, 1:5, 1:1, 5:1, and 10:1 (platelet: microorganism). Finally, they were analyzed by flow cytometry (no centrifugation was used after platelet–microorganism contact).

### 4.4. Antimicrobial Molecules Secreted by Platelets in Response to LL-37

Supernatants collected from platelets treated with LL-37 or the control condition (Section 4.2) and the secretion of BPI, MPO, HNP1 (R & D System), and Azu (Sino Biological, Beijing, China) were quantified by ELISA (enzyme-linked immunoadsorbent assay) using an optical density. A capture antibody was used for each antimicrobial molecule and incubated overnight. Subsequently, blocking was performed using bovine albumin (BSA) (Sigma-Aldrich, St. Louis, MO, USA) for one hour. Next, 100 µL of the sample was placed in each well per condition and left to incubate for two hours. Afterwards, the wells were washed with PBS buffer (Phosphate buffered saline) three times, and a detection antibody was added, followed by a secondary antibody coupled to HRP (rabbit peroxidase), according to the instructions from each supplier of the ELISA Kits. Readings were performed at 450 nm using a microplate reader (Model 4300, Awareness Technology Inc. Chromate, Palm City, FL, USA) with the Leica Suite 345 X software application (LAS-X, Leica-Microsystems, Wetzlar, Germany).

### 4.5. Antimicrobial Activity Assay

The broth microdilution method evaluated the antimicrobial properties of the culture supernatant of resting and LL37-activated platelets (10 or 20 µM) in vitro against the Gram-negative bacterium *E. coli.* From the microbial inoculum grown in Müller–Hinton broth, a triplicate dilution series of the supernatant of LL37-activated platelets was aseptically prepared in a 96-well microtiter plate. Each well was inoculated with 100 μL of a microbial suspension adjusted to a final optical density (OD) of 0.5 McFarland units. The microplate was incubated under aerobic conditions under shaking (120 rpm) at 37 °C for 20 h in a microorganism incubator (Model 9082, ECOSHEL, Denver, CO, USA) Subsequently, 100 μL of the platelet supernatant was placed in each well or LL-37 (10 or 20 µM), and serial dilutions were performed. Microbial growth was detected by measuring the OD at 630 nm using an ELISA microplate reader (Section 4.4).

Microbial growth was measured by microdilution plate assay and optical density, as described in this section. Platelets purified according to Section 4.1 were activated with thrombin (2 units) or went without stimulus for 30 min at 30 °C and 5% CO_2_. The supernatant obtained from activated platelets was treated with 5µg/mL of an isotype IgG mAb (IgG1 κ, Mouse, mAb MOPC-21) or the anti-LL-37 mAb (LL-37/CAP-18, Human, clone 3D11 Cat. number HM2070-100UG), both manufactured by Hycult Biotech Inc. (987 Old Eagle Road, Wayne, PA, USA) for 30 min at 5 °C, before contacting *E. coli*, *S. aureus*, and *C. Albicans* for 13 h.

### 4.6. Platelet Translation Assays

Peripheral blood platelets were obtained as described in Section 4.1 and cultured at a concentration of 5 × 10^7^ cells/mL in RPMI (Roswell Park Memorial Institute medium) supplemented with 10% SFB (fetal bovine serum). In some conditions, platelets received pretreatment with 8 µg/mL of puromycin (Sigma-Aldrich, MO, USA), and 30 min later received or did not receive stimulation with 10 µM of LL-37. Incubation times were 0.5 and 9 h at 37 °C and 5% CO_2_. After incubation, the total protein (cellular and secreted) was obtained by centrifuging the tubes of each condition at 3500× *g* for 10 min at 4 °C, recovering the supernatant and lysing the cell button, leaving 30 min of incubation with a RIPA (Radioimmunoprecipitation assay) buffer. Subsequently, centrifugation was performed at 15,000 *g* for 15 min at 4 °C to obtain the total protein. Finally, Azu and MPO concentrations were determined by ELISA (Section 4.4).

## 5. Conclusions

The results demonstrate that LL-37 treatment increases different antimicrobial functions in human platelets, which correspond to (1) expression of receptors for direct and indirect recognition of microorganisms (TLR-2 and -4, CD32, CD206, Dectin-1, CD35, LOX-1, CD41, CD62P, and αIIbβ3 integrins), as well as molecules related to antigen presentation to T lymphocytes (CD80, CD86, and HLA ABC); (2) platelet binding to *S. aureus* and, to a lesser extent, mediated to *E. coli* and *C. Albicans*; (3) the scretion of antimicrobial molecules: BPI, MPO, HNP1, and Azu; (4) LL-37-treated platelets’ supernatant has an antibacterial activity against E. coli; and (5) the activation of Azu translation. Finally, we demonstrate that platelet LL-37 exhibits antimicrobial activity against *E. coli*, *S. aureus*, and *C. albicans*. However, it is essential to block or use a modified LL-37 to discuss the effect of LL-37 on platelet antimicrobial activity and to know the impact of each molecule increased on the surface of platelets or secreted by platelets through the effects of LL-37, as outlined in this work.

## Data Availability

The data presented in this study are available on request from the corresponding author.

## Supplementary Materials

The following supporting information can be downloaded at: https://www.mdpi.com/article/10.3390/ijms24032816/s1.
