# Supplementary material for: LL-37 Triggers Antimicrobial Activity in Human Platelets
Source: Int J Mol Sci. 2023 Feb 1;24(3):2816. doi: 10.3390/ijms24032816 (PMC9917488; doi:10.3390/ijms24032816)
Supplement: Supplementary file 1 [file ijms-24-02816-s001.zip › ijms-2168707-supplementary.pdf]

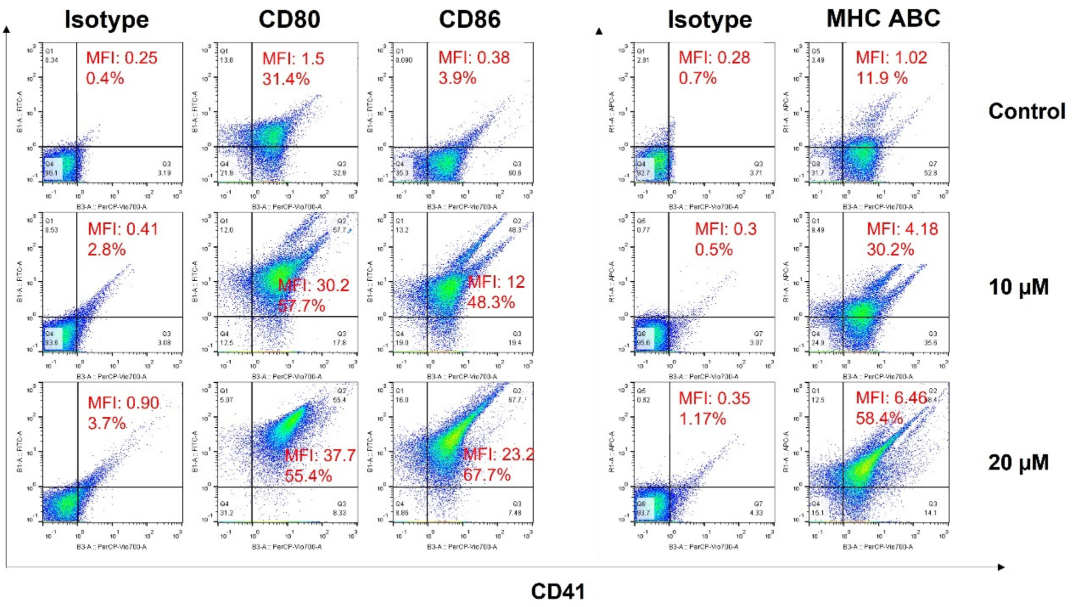

Figure S1. Platelets treated with LL-37 increase the surface expression of molecules associated with T lymphocyte activation. Platelets were purified according to section 4.1 and stimulated with 10 or 20 M LL-37 or without stimuli for 30 minutes at 36 °C and 5% CO2. Subsequently, double labeling was performed using mAbs against CD41 (platelet marker) and CD80, CD86, or MHC ABC and analyzed by flow cytometry. The dot plots correspond to a representative experiment in which the percentage of positive platelets in the upper right quadrant is indicated, and the MFI of total CD41+ platelets was evaluated for each marker.
